# Differential Effect of Dietary Supplementation with a Soybean Oil Enriched in Oleic Acid versus Linoleic Acid on Plasma Lipids and Atherosclerosis in LDLR-Deficient Mice

**DOI:** 10.3390/ijms23158385

**Published:** 2022-07-29

**Authors:** Zhi-Hong Yang, Kimball Nill, Yuki Takechi-Haraya, Martin P. Playford, David Nguyen, Zu-Xi Yu, Milton Pryor, Jingrong Tang, Krishna Vamsi Rojulpote, Nehal N. Mehta, Han Wen, Alan T. Remaley

**Affiliations:** 1 Lipoprotein Metabolism Section, Translational Vascular Medicine Branch, National Heart, Lung and Blood Institute (NHLBI), National Institutes of Health (NIH), 10 Center Drive MSC 1666, Bethesda, MD 20892, USA; haraya@nihs.go.jp (Y.T.-H.); pryorm@nhlbi.nih.gov (M.P.); tangj@mail.nih.gov (J.T.); rojulpoteks@nhlbi.nih.gov (K.V.R.); alan.remaley@nih.gov (A.T.R.); 2 Minnesota Soybean Research & Promotion Council, 1020 Innovation Lane, Mankato, MN 56001, USA; knill@mnsoybean.com; 3 Division of Drugs, National Institute of Health Sciences, 3-25-26 Tonomachi, Kawasaki-ku, Kawasaki 210-9501, Japan; 4 Section of Inflammation and Cardiometabolic Diseases, Cardiovascular Branch, NHLBI, NIH, Bethesda, MD 20892, USA; playfordmp@nhlbi.nih.gov (M.P.P.); nehal.mehta@nih.gov (N.N.M.); 5 Laboratory of Imaging Physics, NHLBI, NIH, Bethesda, MD 20892, USA; davidtn2510@yahoo.com (D.N.); wenh@nhlbi.nih.gov (H.W.); 6 Pathology Core, NHLBI, NIH, Bethesda, MD 20892, USA; yuz@nhlbi.nih.gov

**Keywords:** modified soybean oil, polyunsaturated fatty acids (PUFA), n-6/n-3 PUFA ratio, monounsaturated fatty acids (MUFA), atherosclerosis, inflammation

## Abstract

Both monounsaturated fatty acids (MUFAs) and polyunsaturated fatty acids (PUFAs) play important roles in lipid metabolism, and diets enriched with either of these two fatty acids are associated with decreased cardiovascular risk. Conventional soybean oil (CSO), a common food ingredient, predominantly contains linoleic acid (LA; C18:2), a n-6 PUFA. Recently, a modified soybean oil (MSO) enriched in oleic acid (C18:1), a n-9 MUFA, has been developed, because of its improved chemical stability to oxidation. However, the effect of the different dietary soybean oils on cardiovascular disease remains unknown. To test whether diets rich in CSO versus MSO would attenuate atherosclerosis development, LDL receptor knock-out (LDLR-KO) mice were fed a Western diet enriched in saturated fatty acids (control), or a Western diet supplemented with 5% (*w*/*w*) LA-rich CSO or high-oleic MSO for 12 weeks. Both soybean oils contained a similar amount of linolenic acid (C18:3 n-3). The CSO diet decreased plasma lipid levels and the cholesterol content of VLDL and LDL by approximately 18% (*p* < 0.05), likely from increased hepatic levels of PUFA, which favorably regulated genes involved in cholesterol metabolism. The MSO diet, but not the CSO diet, suppressed atherosclerotic plaque size compared to the Western control diet (Control Western diet: 6.5 ± 0.9%; CSO diet: 6.4 ± 0.7%; MSO diet: 4.0 ± 0.5%) (*p* < 0.05), independent of plasma lipid level changes. The MSO diet also decreased the ratio of n-6/n-3 PUFA in the liver (Control Western diet: 4.5 ± 0.2; CSO diet: 6.1 ± 0.2; MSO diet: 2.9 ± 0.2) (*p* < 0.05), which correlated with favorable hepatic gene expression changes in lipid metabolism and markers of systemic inflammation. In conclusion, supplementation of the Western diet with MSO, but not CSO, reduced atherosclerosis development in LDLR-KO mice independent of changes in plasma lipids.

## 1. Introduction

Dietary fatty acids play significant roles in lipoprotein metabolism and inflammation, a key step in the development of atherosclerosis [1], but their effects differ depending on the type of fatty acid [2]. On balance, saturated fatty acids appear to be pro-atherogenic [3], whereas the consumption of monounsaturated fatty acids (MUFAs) and polyunsaturated fatty acids (PUFAs) is generally associated with less atherosclerotic cardiovascular disease (CVD), a major worldwide cause of morbidity and mortality [4,5,6].

In 2021, the American Heart Association Dietary Guidance for cardiovascular health recommended the replacement of saturated fatty acids with unsaturated fatty acids, especially PUFAs [7]; however, even among PUFAs there are differences in their association with CVD. Numerous epidemiologic studies have shown an inverse association between diets enriched in n-3 PUFA from plants and marine sources with CVD [8]. Dietary linolenic acid (C18:3 n-3), an abundant n-3 PUFA in plant oils, is generally viewed as especially beneficial, because it is an essential precursor of eicosapentaenoic acid (EPA; C20:5 n-3) and docosahexaenoic acid (DHA; C22:6 n-3) [9]. Purified EPA, which has been developed into a drug (Vascepa), lowered serum triglycerides and reduced CVD events when used in conjunction with statins in the REDUCE-IT trial [10]. The cardioprotective effects of n-3 PUFA are likely multifactorial, due to their diverse effects on lipid metabolism, thrombosis, and inflammation. For example, several specialized pro-resolving lipid mediators are derived from n-3 PUFA and have been shown to have potent anti-inflammatory effects [11,12].

Linoleic acid (LA; C18:2 n-6) accounts for approximately 90% of n-6 PUFA intake in the U.S. diet and has also been generally thought to be cardioprotective. The Minnesota Coronary Experiment (MCE) conducted in the 1960s first showed that replacing saturated fat with LA-rich vegetable oils lowered serum cholesterol [13]. In a follow-up study, involving more than 126,000 subjects, higher intake of LA was associated with lower risk for coronary heart disease [14]. A recent re-evaluation of the MCE study failed, however, to find beneficial effects of LA consumption on CVD risk or other health outcomes [15]. Furthermore, several epidemiological and interventional studies found limited evidence for a cardioprotective benefit from dietary n-6 PUFA [16,17]. Although both n-3 and n-6 PUFAs may lower serum lipids, they may have opposing effects in regard to inflammation [18]. Unlike n-3 PUFAs, LA can be converted to arachidonic acid (C20:4 n-6), a precursor for pro-inflammatory lipid mediators such as leukotrienes and prostaglandins [19]. Until the last century, the n-6/n-3 PUFA ratio in most human diets was approximately 1:1 [20,21], but in many economically advanced countries, it is now over 15:1 due to a markedly increased consumption of LA in processed foods containing soybean oil [22]. Soybean oil is highly enriched in LA (>50% of total fatty acids) and now accounts for ~7% of daily calories consumed in the US [23]. Recently, soybeans have been modified by either conventional plant breeding or by genetic engineering to have reduced LA content (1~8% of total fatty acids) and to instead contain higher concentrations of oleic acid (C18:1 n-9). These so-called high-oleic soybean oils have several practical advantages over regular soybean oils in regard to their oxidative stability, sensory taste profiles, and in improving the nutrition profile of animal feeds [24].

Despite its possible widespread public health impact, the effect of consuming conventional soybean oil (CSO) enriched in LA versus a modified soybean oil (MSO) enriched in oleic acid on the development of cardiovascular disease has not been investigated. In the present study, we fed low-density lipoprotein receptor knock-out (LDLR-KO) mice, a Western diet supplemented with either CSO or MSO. LDL-KO mice have been used extensively as animal models of atherogenesis, and they develop lesions in a time-dependent manner, in conjunction with inflammation when placed on a high-fat diet [25]. Our previous study also showed a concurrent increase in plasma lipid levels and inflammation in LDLR-KO mice on a Western diet for 12 weeks [26]. In the current study, we show that although the LA-rich CSO diet lowered plasma lipids better than the MSO diet, the MSO diet decreased atherosclerosis in LDLR-KO mice compared to the control Western diet and CSO diets.

## 2. Results

### 2.1. Fatty Acid Composition of Diets

**Figure 1:**
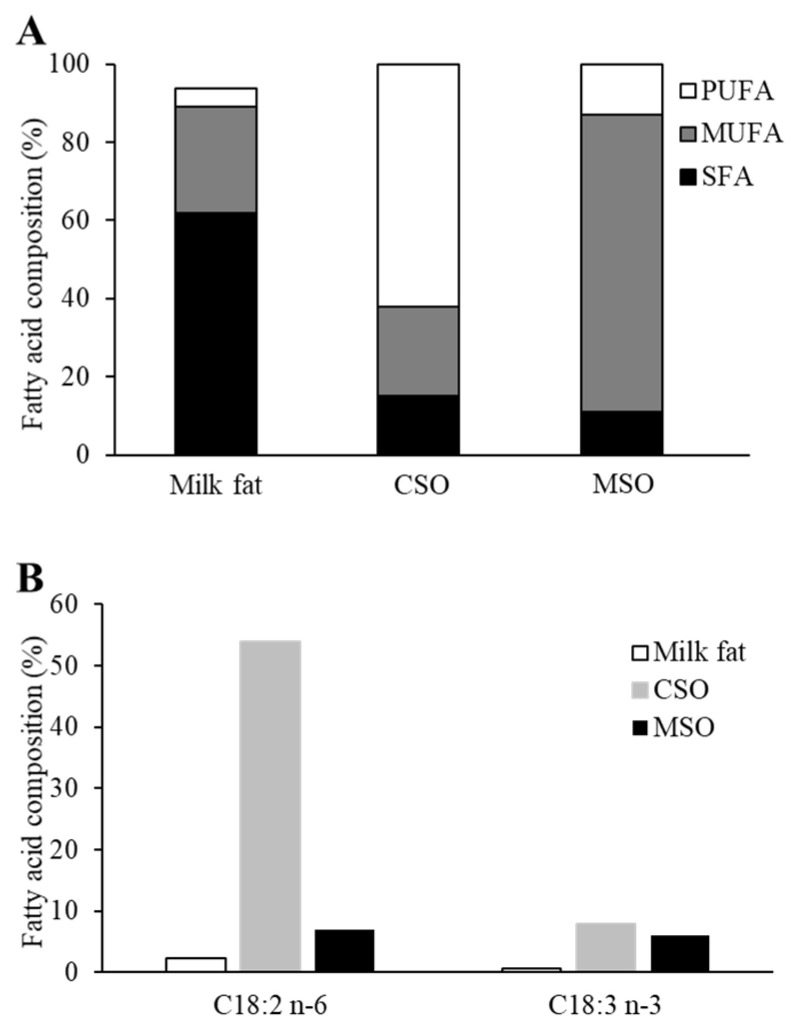
Fatty acid composition of dietary oils incorporated into mouse diets. (**A**) Comparison of total SFA, MUFA and PUFA in the milkfat in Western diet, CSO, and MSO. (**B**) Comparison of PUFA linoleic acid (C18:2 n-6) and linolenic acid (C18:3 n-3) in the milkfat, CSO, and MSO. SFA: saturated fatty acids, MUFA: monounsaturated fatty acid, PUFA: polyunsaturated fatty acid, CSO: conventional soybean oil, MSO: modified soybean oil.

The fatty acid composition of the milk fat used in the control Western diet, as well as the two supplemented soybean oils, CSO and MSO, are shown in Figure 1. The predominant fatty acids in the milk fat were saturated fatty acids (62% of total fatty acids). PUFAs were the main fatty acids in CSO (62% of total fatty acids), whereas MUFAs were the most abundant type of fatty acids in MSO (76% of total fatty acids) (Figure 1A). In regards to their PUFA composition, CSO had a similar percentage of linolenic acid as MSO but had much more LA, which made up about half of the total fatty acids. In contrast, LA was only 7% of the total fatty acids in MSO and was replaced with oleic acid (C18:1 n-9), which resulted in n-6/n-3 PUFA ratio of 1.2 in MSO versus 6.8 for the CSO (Figure 1B). The milk fat enriched in the saturated fatty acid present in the Western diet was partially (5%, *w*/*w*) replaced with either the CSO or MSO to produce three different isocaloric diets that had approximately the same percentage of total protein, carbohydrate, and fat content but different types of fatty acids present (Supplemental Table S1). Mice fed ad libitum showed a similar weight gain over time on the three different diets (Supplemental Figure S1).

### 2.2. Hepatic Fatty Acid Composition

Dietary supplementation with the three different diets significantly affected the fatty acid composition of hepatic lipids (Table 1). The CSO diet increased hepatic total n-6 PUFA compared with the control Western and MSO diet group, largely due to increases in LA and arachidonic acid, the two major n-6 PUFAs in the liver (*p* < 0.05). In contrast, the MSO diet increased oleic acid levels significantly compared with the control Western and CSO diet groups (*p* < 0.05). Hepatic levels of total n-3 PUFA, including linolenic acid, EPA and DHA, significantly increased in both the CSO and MSO diet groups compared to the control group (*p* < 0.05), consistent with the higher content of linolenic acid in the two tested soybean oils compared to the Western control diet enriched in saturated milk fat. Although the hepatic total n-3 PUFA was comparable between the CSO and MSO diet group, the EPA levels in the MSO diet group were two fold higher than in the CSO diet group (*p* < 0.05). The overall percentage of the PUFA content in the liver of the CSO diet group was 169% and 103% higher than that in the control Western diet and MSO diet groups, respectively (*p* < 0.05), and the mice in the MSO diet group had 32.2% higher total hepatic PUFA levels compared with the control Western diet group (*p* < 0.05). Furthermore, the hepatic n-6/n-3 PUFA ratio of mice in the MSO diet group was significantly lower, by 36%, than the control Western diet, and 52% lower than in the CSO diet groups (*p* < 0.05).

### 2.3. Plasma and Hepatic Lipid Profile

**Figure 2:**
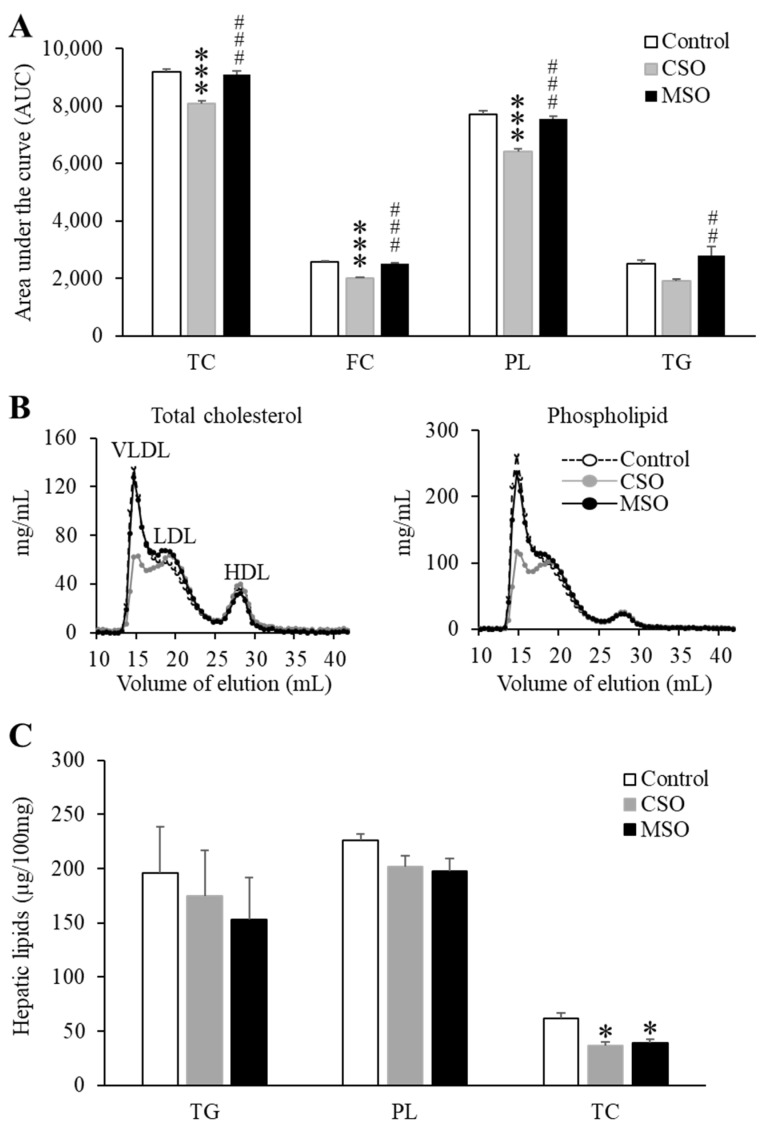
Effects of dietary CSO and MSO on plasma and hepatic lipid profiles in LDLR-KO mice. Mice (n = 12/group) were fed a Western diet supplemented with 5% (*w*/*w*) CSO, MSO, or none (control) for 12 weeks. (**A**) Area under the curve (AUC) for plasma triglyceride, phospholipid, total cholesterol, and free cholesterol from baseline through 12-week period. (**B**) Distribution of pooled plasma total cholesterol and phospholipid in VLDL, LDL and HDL fractions separated by FPLC. (**C**) Hepatic triglyceride, phospholipid, and total cholesterol levels. CSO: conventional soybean oil, MSO: modified soybean oil. TC: total cholesterol, FC: free cholesterol, PL: phospholipid, TG: triglyceride. Values represent the mean ± SEM. * *p* < 0.05, *** *p* < 0.001 compared with the control Western diet; ^##^ *p* < 0.05, ^###^ *p* < 0.001 compared with the CSO diet.

The CSO diet, but not the MSO diet, modestly decreased plasma lipid levels compared to the control diet throughout the 12-week feeding period (Table 2). At the end of the 12-week diet period, plasma phospholipids and free cholesterol levels in the CSO diet were decreased by approximately 10.2% and 21%, respectively, compared to control Western diet (*p* < 0.05). This can also be seen in Figure 2A, where the area-under-curve (AUC) for plasma lipids during the 12-week feeding period is plotted. The CSO diet group lowered the plasma AUC curve for triglyceride, phospholipid, total cholesterol, and free cholesterol by approximately 28%, 15.9%, 11.7%, and 21.2%, respectively, compared to the Western control or the MSO diet group (*p* < 0.05). FPLC profiles show that the reduction in total plasma cholesterol and phospholipids on the CSO diet was mainly due to a reduction in lipids on VLDL and to a lesser degree on LDL (Figure 2B). HDL lipid levels did not appear to change by supplementation with either soybean oil. In addition, we examined whether Western diet-induced hepatic steatosis might be ameliorated by CSO or MSO supplementation. Both CSO and MSO diets modestly decreased hepatic levels of cholesterol by approximately 35% compared with the control Western diet (*p* < 0.05) (Figure 2C). Similar trends were seen for hepatic triglyceride and phospholipid levels but did not reach statistical significance.

### 2.4. Quantification of Aortic Atherosclerosis

**Figure 3:**
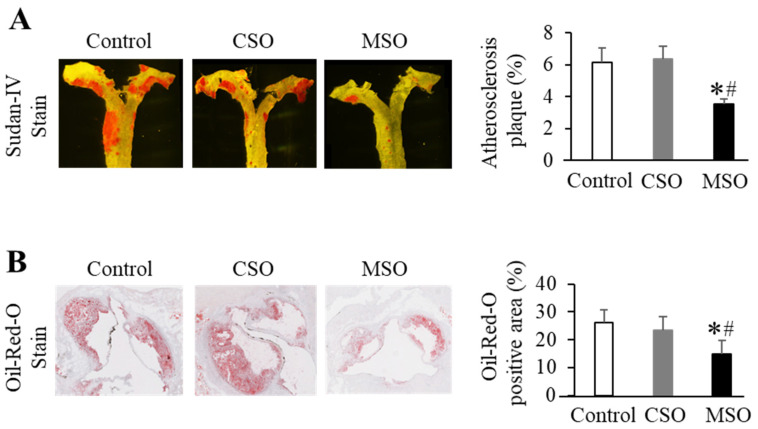
Effects of dietary CSO and MSO on progression of atherosclerosis in LDLR-KO mice. Mice (n = 12/group) were fed a Western diet supplemented with 5% (*w*/*w*) CSO, MSO, or none (control) for 12 weeks. (**A**) Representative *en* *face* Sudan IV staining of aorta (**left**) and quantitative analysis of Sudan IV-positive plaque area of aorta (**right**). (**B**) Representative Oil-Red O staining of aorta sinus **(left**) and quantitative analysis of cross-sectional Oil Red O-positive area of aortic sinus (**right**). CSO: conventional soybean oil, MSO: modified soybean oil. Values represent the mean ± SEM. * *p* < 0.05 compared with the control Western diet; ^#^ *p* < 0.05 compared with the CSO diet.

En face Sudan IV staining of an opened aorta section revealed that mice in the MSO diet group had on average about 39% less atherosclerotic lesion area than those in the control Western and CSO diet groups (*p* < 0.05). No significant differences in lesion area were observed between the control and CSO diet groups (Figure 3A and Supplemental Figure S2). The Oil-Red O staining of cross-sections of the aortic sinus further confirmed the effect of the MSO diet on atherosclerosis development. The Oil-Red stained plaque area in the MSO diet group was approximately 40% less compared to the control Western and CSO diet groups (*p* < 0.05). Again, no differences were observed in lesion area between the control and CSO diet groups (Figure 3B and Supplemental Figure S3). In a small pilot study of mice on the diets for 12 months, atherosclerosis lesion area in the MSO diet group was also less than those in the control or CSO diet groups (*p* < 0.05) (Supplemental Figure S4A,B). There was also a trend toward lower calcification in the aortas of mice on the MSO diet compared with those on the control Western diet (Supplemental Figure S4C).

### 2.5. Plasma Cytokine Levels

**Figure 4:**
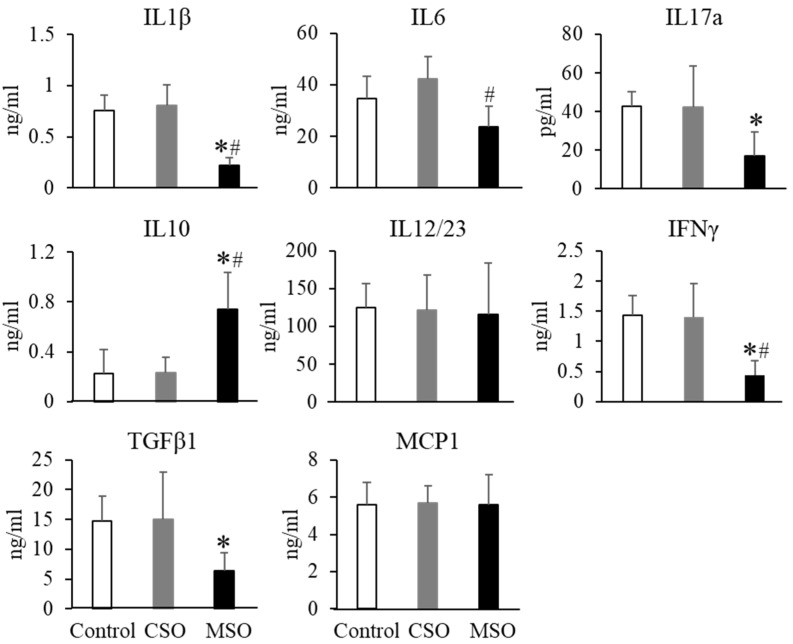
Effects of dietary CSO and MSO on LPS-induced changes in plasma cytokine levels in LDLR-KO mice. Mice (n = 12/group) were fed a Western diet supplemented with 5% (*w*/*w*) CSO, MSO, or none (control) for 12 weeks. CSO: conventional soybean oil, MSO: modified soybean oil. Values represent the mean ± SEM. * *p* < 0.05 compared with the control Western diet; ^#^ *p* < 0.05 compared with the CSO diet.

LPS injection remarkably upregulated proinflammatory cytokine levels in circulation (Figure 4). Feeding with the MSO diet significantly reduced the plasma levels of the pro-inflammatory plasma cytokines following LPS challenge as evidenced by drops in IL1β, IL6, Il17a, IFNγ, and TGFβ1, of 72%, 38%, 60%, 70%, and 57%, respectively, compared to the control Western or CSO diet groups (*p* < 0.05). In contrast, the anti-inflammatory cytokine IL10 levels were increased by 2.2-fold in the MSO diet group compared to the control Western or CSO diet groups (*p* < 0.05). We also observed a more subtle decrease in plasma pro-inflammatory cytokines for mice on the MSO diet compared to the control diets without the LPS induction (Supplemental Figure S5).

### 2.6. Gene Expression in Liver and White Adipose Tissue

**Figure 5:**
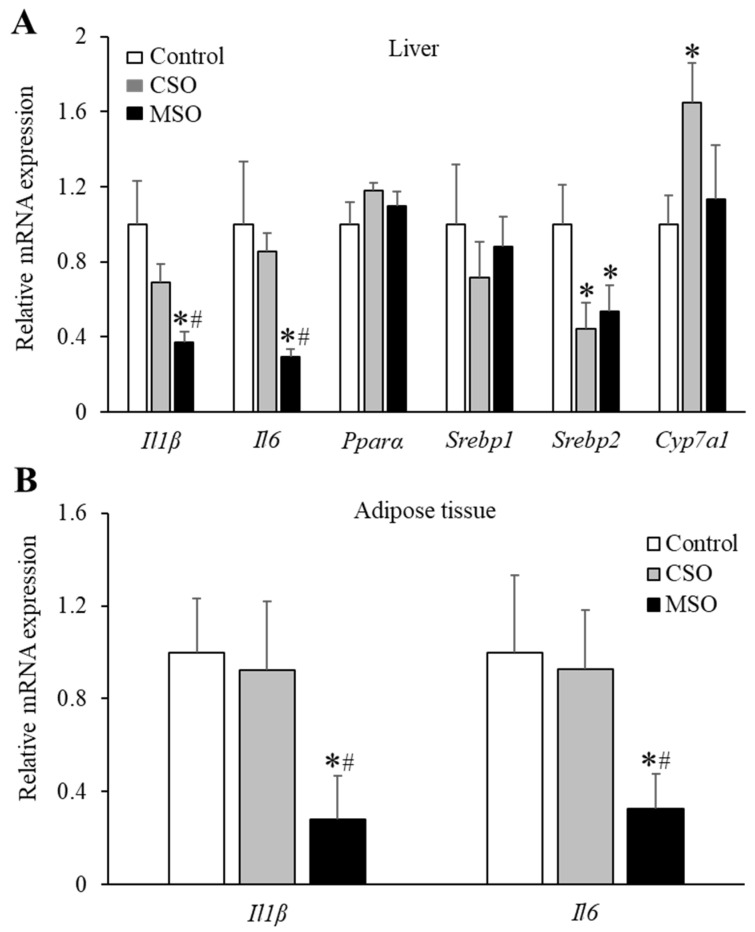
Effects of dietary CSO and MSO on gene expression in LDLR-KO mice. Mice (n = 12/group) were fed a Western diet supplemented with 5% (*w*/*w*) CSO, MSO, or none (control) for 12 weeks. (**A**) Liver mRNA expression of genes involved in inflammation (*Il1β* and *Il6*) and lipid metabolism (*Pparα*, *Srebp1*, *Srebp2*, and *Cyp7a1*). (**B**) mRNA expression of genes related to inflammation (*Il1β* and *Il6*) in white adipose tissue. CSO: conventional soybean oil, MSO: modified soybean oil. Values represent the mean ± SEM. * *p* < 0.05 compared with the control Western diet; ^#^ *p* < 0.05 compared with the CSO diet.

To test whether the expression of genes involved in inflammation and lipid metabolism can be differentially modulated by supplementation with the two different soybean oils, we conducted RT-PCR analysis in liver and in white adipose tissue. As shown in Figure 5, the MSO diet significantly downregulated mRNA expression of inflammatory cytokine genes *Il1β* and *Il6* in the liver and white adipose tissues, compared to the control Western or CSO diet groups. Supplementation of CSO or MSO also decreased *Srebp2* mRNA levels in the liver compared with the control Western diet (*p* < 0.05). CSO diet, but not the MSO diet, also increased hepatic *Cyp71a* mRNA expressions compared with control Western diet (*p* < 0.05). No major differences in *Pparα* mRNA levels were observed among the three diet groups.

## 3. Discussion

In this study, we evaluated the effects of two types of soybean oils with different fatty acid profiles on atherosclerosis development in mice fed a Western diet. The milk fat used in the Western control diet is high in saturated fatty acids, which has been shown to promote the development of aortic atherosclerosis in LDLR-KO mice, partly through upregulating inflammatory signaling [27]. The saturated fatty acids in the Western diet were partially replaced with the two different soybean oils that contained comparable amount of saturated fatty acids and n-3 linolenic acid but differed in their content of unsaturated fatty acid types, i.e., n-6 PUFA and MUFA, as well as the n-6/n-3 PUFA ratios. Compared to the control Western diet, supplementation with the LA-rich CSO, but not the MUFA-rich MSO diet, consistently reduced plasma lipid levels and lowered cholesterol in VLDL and LDL. In agreement with our results, previous studies in humans have demonstrated that increased LA consumption reduces blood cholesterol levels, and that a higher dietary PUFA/MUFA ratio also leads to lower plasma lipid levels [28,29]. Previous studies have also shown that both unmodified soybean oil and high-oleic specialty soybean oil improved lipoprotein profiles in moderately hyperlipidemic subjects compared with partially hydrogenated fatty acids [30], suggesting that they can be used for reducing trans fatty acid exposure. The lipid-lowering effects of LA are generally thought to occur through improved hepatic clearance of circulating LDL driven by enhanced transcription of nuclear receptors, such as PPARs and LXR [31]. In addition, SREBPs, key hepatic transcription factors for regulating triglyceride and cholesterol metabolism, are also involved in this process [32]. In the current study, the CSO diet reduced hepatic cholesterol content compared to the control Western diet. The plasma and hepatic cholesterol-lowering effect may be partly due to down-regulating hepatic mRNA expression of *Srebp2* and up-regulating *Cyp7a1*, which encodes a key enzyme for the conversion of cholesterol into bile acids [33]. Although the MUFA-rich MSO diet did not lower plasma lipids, it did decrease the hepatic cholesterol content compared with the control Western diet, possibly due to reduced *Srebp2* gene expression. The hepatic content of linolenic acid and total n-3 PUFA in both plant oil diet groups increased to the same extent, which may be responsible for their similar reduction in hepatic cholesterol contents. In previous studies, when linolenic-rich plant oil was supplemented in high-fat diet, overall lipid metabolism was improved in rodents, which was attributed to the reduction in cholesterol synthesis in liver tissues [34]. Thus, our data shows that the total amount of PUFA, rather than n-6/n-3 PUFA ratio correlated with the beneficial effect of CSO in blood lipids by possibly regulating the hepatic expression of genes involved in lipid metabolism.

A quantification of the lesion area of the aortic sinus and whole aortic plaques revealed a significant decrease in lesion size in the MSO diet group enriched in oleic acid compared to the control Western or CSO diet groups, despite the greater ability of the CSO diet to reduce plasma lipid levels. Based on a substantial body of epidemiologic data showing negative associations between a MUFA-rich diet and coronary heart disease risk, the American Heart Associate has recommended replacing saturated fatty acids with MUFAs [35,36], which are commonly found in the so-called Mediterranean diet. In addition to oleic acid, the MSO used in our study has a considerable amounts of linolenic acid with a n-6/n-3 PUFA ratio of approximately 1:1. The CSO diet contains a similar amount of linolenic acid but with a n-6/n-3 PUFA ratio of ~8:1. It has been shown in animal models that a lower n-6/n-3 PUFA ratio in the diet may be cardioprotective by improving inflammatory status and endothelial function [37]. Furthermore, human intervention studies showed that the plasma and tissue n-6 and n-3 PUFA profiles could be modified and improved by consuming low-LA diet or diets enriched in n-3 PUFAs [38,39]. In our study, consumption of the CSO diet resulted in higher hepatic LA and arachidonic acid levels compared with the MSO diet group. Arachidonic acid competes with the metabolism of EPA and is also a direct precursor for a variety of pro-inflammatory mediators, such as prostaglandins, thromboxanes, and leukotrienes [40]. Excess hepatic uptake of n-6 PUFA may, therefore, promote the formation of proinflammatory oxidized LA metabolites, such as 9-hydroxy-octadecadienoic acid [41].

In regards to the hepatic n-3 PUFA content, despite a similar linolenic acid content, the MSO diet resulted in a more efficient EPA synthesis from linolenic acid compared with the CSO diet. There were no differences, however, in hepatic DHA levels between the two soybean oil diet groups possibly due to an inefficient conversion of linolenic acid to DHA [42]. LA and linolenic acid are precursors for the longer chain n-6 and n-3 fatty acids, respectively, and are competitively metabolized by two non-interconvertible pathways involving the same enzymes. Therefore, when n-3 linolenic acid and n-6 LA are both consumed, they compete for incorporation into cell membranes. A LA-rich diet may, therefore, result in the replacement of EPA in cell membrane by arachidonic acid that is converted from LA. In humans the conversion of linolenic acid to longer-chain n-3 PUFA can be reduced by up to 50% with high LA content in diet [43]. EPA elicits anti-inflammatory actions through multiple mechanisms, including disruption of lipid rafts, reducing expression of inflammatory genes through inhibiting activation of the pro-inflammatory transcription factor nuclear factor kappa B, and producing mediators that inhibit arachidonic acid release from phospholipids [44]. Studies in animal models and human subjects have demonstrated beneficial effects of EPA on reducing atherosclerotic lesions, potentially through its anti-inflammatory effects [45,46]. Although inflammation is critical during the initial activation of the immune system from infections, sustained inflammation can be harmful and is thought to promote atherosclerosis [47]. These previous findings may explain why the MSO diet dampened LPS-induced inflammation in our mouse model, as evidenced by reduced circulating pro-inflammatory cytokines and the downregulation of several cytokine-related genes in the liver, including *Il1β* and *Il6*. We also observed similar anti-inflammatory gene expression changes on the MSO diet in white adipose tissue, an important endocrine organ that releases pro- and anti-inflammatory cytokines from macrophages and other immune cells in fat mass [48]. In contrast to saturated fatty acids, which are potent inducers of pro-inflammatory cytokines expression, MUFAs and n-3 PUFAs are known to suppress LPS-induced production of pro-inflammatory cytokines [49,50]. Furthermore, the MSO diet also increased plasma levels of IL-10, a potent anti-inflammatory cytokine that limits macrophage activation and infiltration [51]. It is noteworthy that although the hepatic oleic acid levels in the control group were significantly higher than that in the CSO group, the control Western diet had the lowest hepatic levels of total n-3 PUFA among the three diet groups, which may have contributed to atherosclerosis development comparable to that in the CSO group. Thus, it may be that it is the favorable balance of the unsaturated fatty acids in the MSO diet, rather than the total amount of n-6 LA, that may account for the observed attenuation of inflammation and atherosclerosis in our mouse model. Limitations of the present study include that only hepatic fatty acid composition was measured, and specific fatty acid species-derived metabolites in circulation and the aorta were not determined. In the future studies, such as those comparing fatty acid composition between the blood, liver, and aorta, evaluating the effect of soybean oil-rich diets on aortic inflammation and the production of proinflammatory mediators derived from n-6 PUFA, are needed to better explore the relationship between the metabolism of different dietary soybean oil types and atherosclerosis and other inflammatory diseases.

In summary, we compared the dietary effects of CSO versus MSO on atherosclerosis development in LDLR-KO mice. In agreement with previous studies, our results showed the lipid-lowering effect of the CSO diet enriched in LA, but only the MSO diet decreased atherosclerosis compared to the Western diet. This finding is likely due to the alteration of the unsaturated fatty acid composition in liver tissue, which may be associated with lower inflammatory biomarkers. Compared with most other commercial high-oleic soybean oils, the novel MSO used in the current study contains a considerable amount of linolenic acid, which not only resulted in the increased total intake of unsaturated fatty acids but may have also dampened inflammation by lowering the n-6/n-3 PUFA ratio. If our findings can be confirmed in human dietary studies, it would suggest that the replacement of conventional soybean oils rich in LA with MSO rich in oleic acid and linolenic acid could be beneficial for cardiovascular disease prevention.

## 4. Materials and Methods

### 4.1. Animals and Experimental Design

Eight-week old LDLR-KO mice were purchased from Jackson Lab (Bar Harbor, ME, USA). High-LA CSO and low-LA MSO (TruSoya^®^ Non-GMO High Oleic Soybean Oil) were provided by the Minnesota Soybean Association (Minnesota). Female LDLR-KO mice were fed a Western diet TD.88137 Adjusted Calories Diet (Harlan Teklad, Madison, WI, USA), supplemented with 5% (*w*/*w*) CSO, 5% (*w*/*w*) MSO, or not (control) for 12 weeks (n = 12/group). Plasma lipids were monitored every 3 weeks in 5 h-fasting animals. At the end of the 12-week feeding period, mice were sacrificed to collect tissue and blood samples for further studies. All animal care and experimental protocols in this study were conducted in accordance with guidelines provided by the NIH Guide for the Care and Use of Laboratory Animals and approved by the Animal Care and Use Committee of the National Heart, Lung and Blood Institute (Protocol #H-0050).

### 4.2. Biochemical Analysis

At the end of the 12-week feeding period, lipoprotein fractions, including very low-density lipoprotein (VLDL), low-density lipoprotein (LDL) and high-density lipoprotein (HDL), were separated by fast protein liquid chromatography (FPLC) from pooled plasma (n = 12/group), using an Akta Pure instrument equipped with two Superose 6 columns (GE Healthcare, Chicago, IL, USA). Triglycerides, phospholipids, total cholesterol, and free cholesterol were measured in plasma and FPLC fractions, using enzymatic test kits from FUJIFILM Wako Chemicals (Richmond, VA, USA). After 12 weeks on the diets, mice received an I.P. injection of 1 mg/kg of lipopolysaccharide (LPS) (Sigma-Aldrich, St. Louis, MO, USA). Retro-orbital bleeding was performed at 4 h post-LPS injection, and the plasma concentrations of cytokines, including interleukin-1β (IL1β), interleukin-6 (IL6), interleukin-17a (IL17a), interleukin-10 (IL10), interleukin-12/23 (IL12/23), monocyte chemoattractant protein-1 (MCP1), and transforming growth factor- β1 (TGFβ1) were measured using a customized multiplex assay system (Uplex, Mesoscale, Gaithersburg, MD, USA).

### 4.3. Hepatic Lipids and Fatty Acid Composition Analysis

Liver lipids were extracted with chloroform/methanol solution (2:1 *v/v*) [52]. Total lipid extracts were dried under nitrogen, and dissolved with ethanol, containing 1% of Triton X-100 for the measurement of triglyceride, phospholipid, and total cholesterol with enzymatic reagent kits (FUJIFILM Wako Chemicals). For hepatic fatty acid composition, lipids were extracted by homogenizing tissue samples in a methanol/hexane solution (4:1 *v/v*) [53]. The hepatic fatty acids were converted to fatty acid methyl esters (FAME) by reaction with acetyl chloride at 100 °C for 1 h, and the resulting FAME were separated and analyzed by gas chromatography on a Shimadzu GC2010 (Shimadzu Scientific Instruments, Columbia, IN, USA) equipped with an FID detector and a capillary DB-FFAP column (30 m  ×  0.32 mm, φ 0.25 μm; Agilent, Santa Clara, CA, USA). Peaks were identified by comparison of retention times of a reference mixture (Nu-Chek Prep. Inc., Elysian, MN, USA). Data were expressed as a percentage of each fatty acid of the total fatty acids in each sample (% wt).

### 4.4. Atherosclerotic Lesion and Aortic Calcification Analyses

At the end of the 12-week feeding period, atherosclerotic lesion development was assessed by en face analysis of the whole aorta [54]. The aorta was briefly fixed with 10% neutral buffered formalin, stained with Sudan IV solution, and the excess stain was washed off with 70% ethanol. After excess fat tissue was removed, the aorta was cut open longitudinally and imaged for lesion area analysis, using ImagePro Premier version 9.1 software (Media Cybernetics, Silver Spring, MD, USA). The aortic plaque lesions were expressed as a percentage of the area of lesions relative to the surface area of the entire aorta. In addition, the frozen aortic root samples were cut into 10-µm cross-sections. Sections were fixed with 10% neutral buffered formalin, washed with PBS, and then treated with 60% isopropanol. Following Oil-Red-O (Sigma) stain and wash with 60% isopropanol, the sections were counterstained with hematoxylin. All slides were then scanned with Hamamatsu NDP scanner for histology evaluations with ImagePro software. The extent of the lipid accumulation was expressed as the area percentage of positive staining. The amount of aortic calcification was quantified with an X-ray microscopy approach as described previously [55]. Briefly, an X-ray tomosynthesis scanner was used to scan the fresh aorta samples, which were fully immersed in de-ionized water, and quantitative images of X-ray absorption were digitally constructed. The density of calcification was measured with a modified single-energy X-ray absorptiometry method.

### 4.5. Gene Expression

Total RNA from liver and white adipose tissue were extracted using a RNeasy kit (Qiagen, Valencia, CA, USA). cDNA synthesis was performed, using a PrimeScript 1st strand cDNA Synthesis Kit (Takara Bio USA, Inc. San Jose, CA, USA). Gene expression was assessed by quantitative reverse transcription (RT)-PCR analysis on an Applied Biosystems 7900 HT Fast Real-Time PCR System (Applied Biosystems, Waltham, MA, USA), using the TaqMan Universal PCR Master Mix (Thermo Fisher Scientific, Waltham, MA, USA). The following TaqMan probes (Thermo Fisher Scientific) were used: mouse *Il1β* (ID: Mm00434228_m1), *Il6* (ID: Mm00446190_m1), *Pparα* (Peroxisome Proliferator Activated Receptor Alpha; ID: Mm00440939_m1), *Cyp7a1* (Cytochrome P450 Family 7 Subfamily A Member 1; ID: Mm00484150_m1), *Srebp1* (sterol regulatory element-binding protein 1; ID: Mm00550338_m1), *Srebp2* (ID: Mm01306292_m1). *β-actin* was used as the housekeeping genes, and gene expression was scaled to the expression of the *β-actin*.

### 4.6. Statistical Analysis

Statistical analyses between the three diet groups were performed using GraphPad Prism version 9.0.2 (GraphPad Software Inc. La Jolla, CA, USA). Data are expressed as the mean ± S.E.M. and were analyzed by a one-way ANOVA followed by a Tukey post hoc test where appropriate. *p* < 0.05 was considered statistically significant.

## Figures and Tables

**Table 1:** Effect of CSO and MSO diets on hepatic fatty acid composition in LDLR-KO mice.

Fatty Acids (%)	Control Western Diet	CSO Diet	MSO Diet
12:0	0.11 ± 0.01	0.12 ± 0.01	0.09 ± 0.01
14:0	1.38 ± 0.05	1.25 ± 0.08	1.17 ± 0.05
16:0	21.82 ± 0.55	22.1 ± 0.22	21.43 ± 0.74
16:1 n-7	4.3 ± 0.26	3.64 ± 0.26	3.9 ± 0.26
18:0	5.64 ± 0.38	5.66 ± 0.29	5.05 ± 0.22
18:1 n-9	39.9 ± 1.02	32.76 ± 0.91 ***	44.53 ± 0.48 ** ^####^
18:1 n-7	2.28 ± 0.13	1.03 ± 0.08 ****	1.11 ± 0.14 ****
18:2 n-6	3.05 ± 0.1	13.04 ± 0.43 ****	4.32 ± 0.5 ^####^
18:3 n-6	0.04 ± 0.001	0.19 ± 0.01 ****	0.06 ± 0.001 ^####^
18:3 n-3	0.09 ± 0.01	0.61 ± 0.04 ****	0.46 ± 0.04 ***
20:0	0.04 ± 0.001	0.05 ± 0.002	0.04 ± 0.001
20:1 n-9	1.21± 0.04	0.47 ± 0.04 ****	1.05 ± 0.05 ^####^
20:2 n-6	0.04 ± 0.002	0.13 ± 0.001 ****	0.05 ± 0.001 * ^####^
20:3 n-6	1.39 ± 0.26	1.56 ± 0.21	0.06 ± 0.11 *^##^
20:3 n-3	N.D.	0.01 ± 0.0001	0.01 ± 0.0001
20:4 n-6	2.74 ± 0.12	3.89 ± 0.19 *	2.83 ± 0.37 ^#^
20:5 n-3	0.06 ± 0.001	0.13 ± 0.01 *	0.27 ± 0.02 **** ^##^
22:0	N.D.	N.D.	0.02 ± 0.001
22:1 n-9	0.1 ± 0.02	0.1 ± 0.06	0.05 ± 0.01
22:5 n-3	0.01 ± 0.0001	0.01 ± 0.001	0.01 ± 0.01
22:6 n-3	1.41 ± 0.01	2.29 ± 0.02 **	2.17 ± 0.02 **
24:0	0.27 ± 0.01	0.18 ± 0.01 **	0.24 ± 0.02 ^#^
24:1 n-9	0.1 ± 0.01	0.18 ± 0.09 ****	0.18 ± 0.01 *
Total SFA	29.27 ± 0.35	29.37 ± 0.36	28.05 ± 0.61
Total MUFA	48.04 ± 1.25	38.29 ± 1.01 ****	50.9 ± 0.73 ^####^
Total PUFA	8.28 ± 0.9	22.28 ± 0.71 ****	10.95 ± 0.93 **** ^###^
Total n-6 PUFA	6.74 ± 0.73	19.08 ± 0.57 ****	8.06 ± 0.72 ^####^
Total n-3 PUFA	1.69 ± 0.11	3.2 ± 0.17 ****	2.88 ± 0.25 ***
n-6/n-3 ratio	4.49 ± 0.23	6.08 ± 0.22 ****	2.87 ± 0.21 **** ^####^

Mice (n = 12/group) were fed a Western diet supplemented with 5% (*w*/*w*) CSO, MSO, or none (control) for 12 weeks. CSO: conventional soybean oil, MSO: modified soybean oil, ND: not detected, SFA: saturated fatty acids, MUFA: monounsaturated fatty acids, PUFA: polyunsaturated fatty acids. Values represent the mean ±SEM. * *p* < 0.05, ** *p* < 0.01; *** *p* < 0.001; **** *p* < 0.0001 compared with the control Western diet; ^#^ *p* < 0.05, ^##^ *p* < 0.01, ^###^ *p* < 0.001, ^####^ *p* < 0.0001, compared with the CSO diet.

**Table 2:** Effect of CSO and MSO diets on plasma lipid levels in LDLR-KO mice.

Lipids	Control	CSO Diet	MSO Diet
Week 0			
TC	251.6 ± 6.5	227.7 ± 21.3	244.5 ± 7.2
FC	84.9 ± 4.2	78.6 ± 2	83.4 ± 2.2
PL	239.1 ± 8.7	219.6 ± 7.9	255.7 ± 8.8
TG	178.3 ± 11.9	156.9 ± 6.2	161.9 ± 6.5
Week 3			
TC	783.8 ± 12.1	737.2 ± 12.6 *	813 ± 13.5 ^###^
FC	211.7 ± 4.6	190.4 ± 6.2 *	211 ± 6.4 ^##^
PL	666 ± 15.8	590.9 ± 17.5 **	675.6 ± 16.9 ^##^
TG	223.3 ± 16.9	219.5 ± 20.7	282 ± 25.2
Week 6			
TC	882.6 ± 13	764.9 ± 13.6 ****	859.6 ± 18.1 ^###^
FC	237.4 ± 3.8	178 ± 5.7 ****	229.9 ± 5.7 ^####^
PL	712.9 ± 11.8	565.4 ± 12.8 ****	684.2 ± 17.1 ^####^
TG	224.1 ± 17	155 ± 19.8	259.0 ± 40.4 ^#^
Week 9			
TC	880.8 ± 13.2	711.4 ± 22.9 ****	839.6 ± 17.3 ^####^
FC	250.6 ± 4.7	166.3 ± 7.5 ****	231.2 ± 6.8 ^####^
PL	711.6 ± 10.8	546.1 ± 16.9 ****	671.7 ± 14.6 ^####^
TG	199.5 ± 15.1	93.7 ± 13.8 *	207.6 ± 37.8 ^##^
Week 12			
TC	780.9 ± 14.6	735.6 ± 171.1	806.7 ± 13.7 ^##^
FC	234.3 ± 13.2	185 ± 7.6 ****	219.2 ± 7.8 ^##^
PL	727 ± 13.2	653.2 ± 27.9 *	708.2 ± 17.9
TG	179.3 ± 20	175.3 ± 26.7	204.1 ± 33.1

Mice (n = 12/group) were fed a Western diet supplemented with 5% (*w*/*w*) CSO, MSO, or none (control) for 12 weeks. CSO: conventional soybean oil, MSO: modified soybean oil. TC: total cholesterol, FC: free cholesterol, PL: phospholipid, TG: triglyceride. Values represent the mean ± SEM. * *p* < 0.05, ** *p* < 0.01; **** *p* < 0.0001 compared with control. ^#^ *p* < 0.05; ^##^ *p* < 0.01; ^###^ *p* < 0.001; ^####^ *p* < 0.0001, compared with CSO diet.

## Data Availability

Data are contained within the article.

## Supplementary Materials

The following supporting information can be downloaded at: https://www.mdpi.com/article/10.3390/ijms23158385/s1.
