# Supplementary material for: Differential Effect of Dietary Supplementation with a Soybean Oil Enriched in Oleic Acid versus Linoleic Acid on Plasma Lipids and Atherosclerosis in LDLR-Deficient Mice
Source: Int J Mol Sci. 2022 Jul 29;23(15):8385. doi: 10.3390/ijms23158385 (PMC9369370; doi:10.3390/ijms23158385)

Supplemental Table S1. Diet compositions

Ingredient	Western diet (Control) (g/kg diet)	CSO diet (g/kg g diet)	MSO diet (g/kg diet)
Casein	195	195	195
DL-Methionine	3	3	3
Sucrose	341.5	341.4	341.4
Corn Starch	150	150	150
Cholesterol	1.5	1.5	1.5
Cellulose	50	50	50
Mineral Mix	35	35	35
Calcium Carbonate	4	4	4
Vitamin Mix	10	10	10
Ethoxyquin, antioxidant	0.04	0.04	0.04
Anhydrous Milkfat	210	160	160
CSO	—	50	—
MSO	—	—	50
	**Nutrient Information (% kcal from)**		
Protein	15.2	15.2	15.2
Carbohydrate	42.7	42.7	42.7
Fat	42	42	42
Kcal/g	4.5	4.5	4.5

CSO: conventional soybean oil, MSO: modified soybean oil.

Supplemental Figure S1. Effects of dietary CSO and MSO on body weight in LDLR-KO mice. Mice 17 (n = 12/group) were fed a Western diet supplemented with 5% (w/w) CSO, MSO, or none (control) 18 for 12 weeks. CSO: conventional soybean oil, MSO: modified soybean oil.

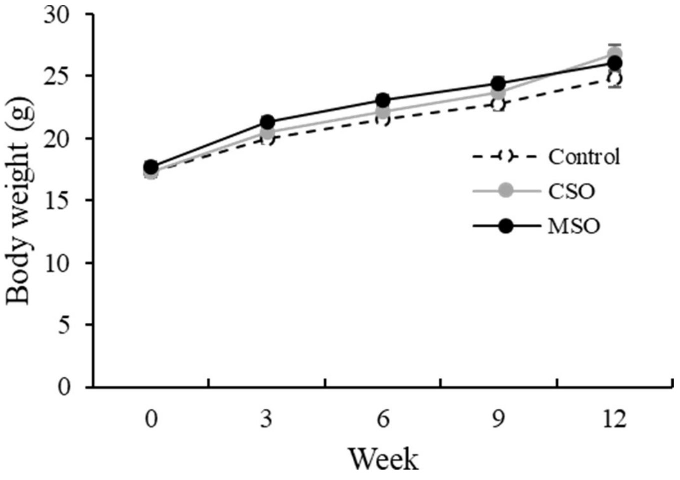

Supplemental Figure S2. En face SUDAN-IV stain of aorta in LDLR-KO mice. Mice (n = 12/group) were fed a Western diet supplemented with 5% (w/w) CSO, MSO, or none (control) for 12 weeks. *En face* SUDAN-IV stain of aorta. CSO: conventional soybean oil, MSO: modified soybean oil.

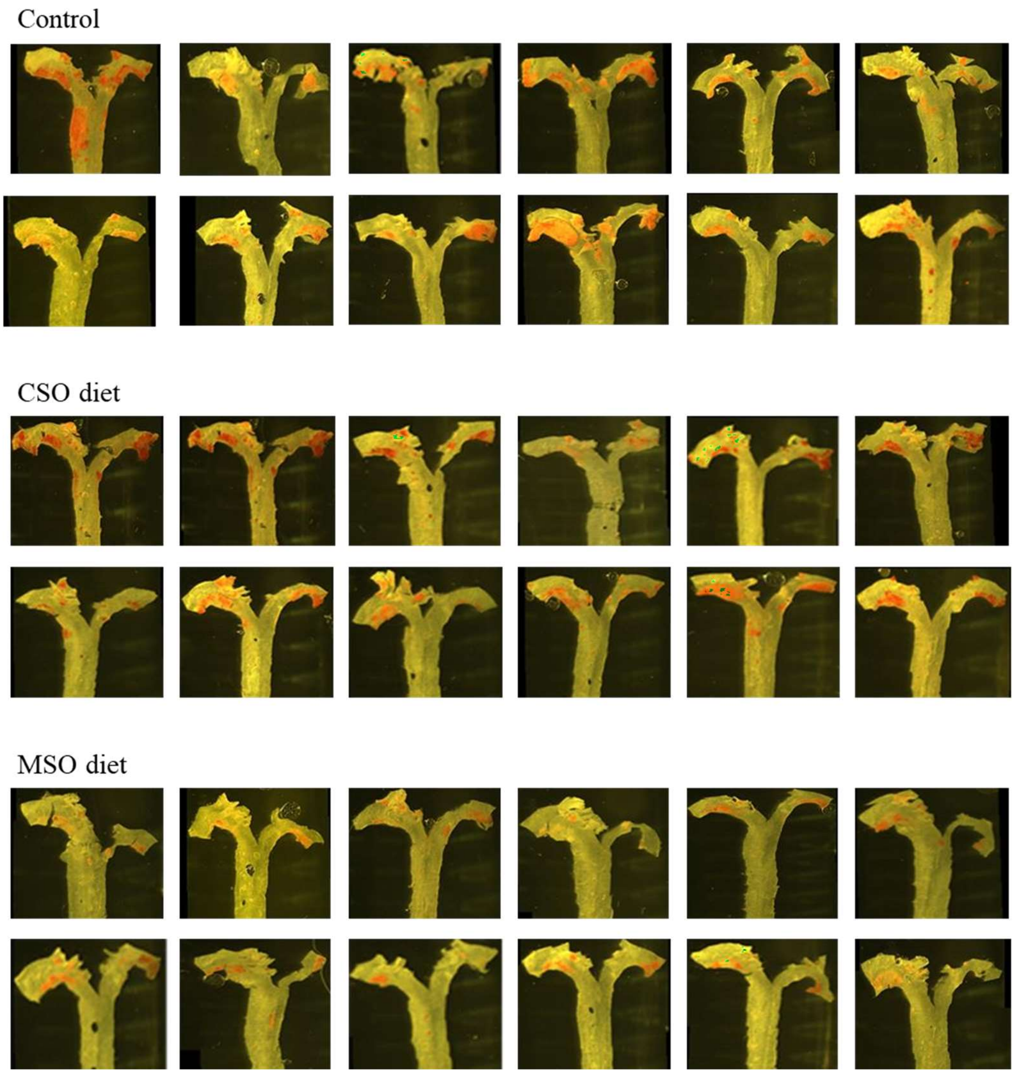

Supplemental Figure S3. Oil-Red O stain of aorta sinus cross-sections in LDLR-KO mice. Mice (n = 12/group) were fed a Western diet supplemented with 5% (w/w) CSO, MSO, or none (control) for 12 weeks. CSO: conventional soybean oil, MSO: modified soybean oil.

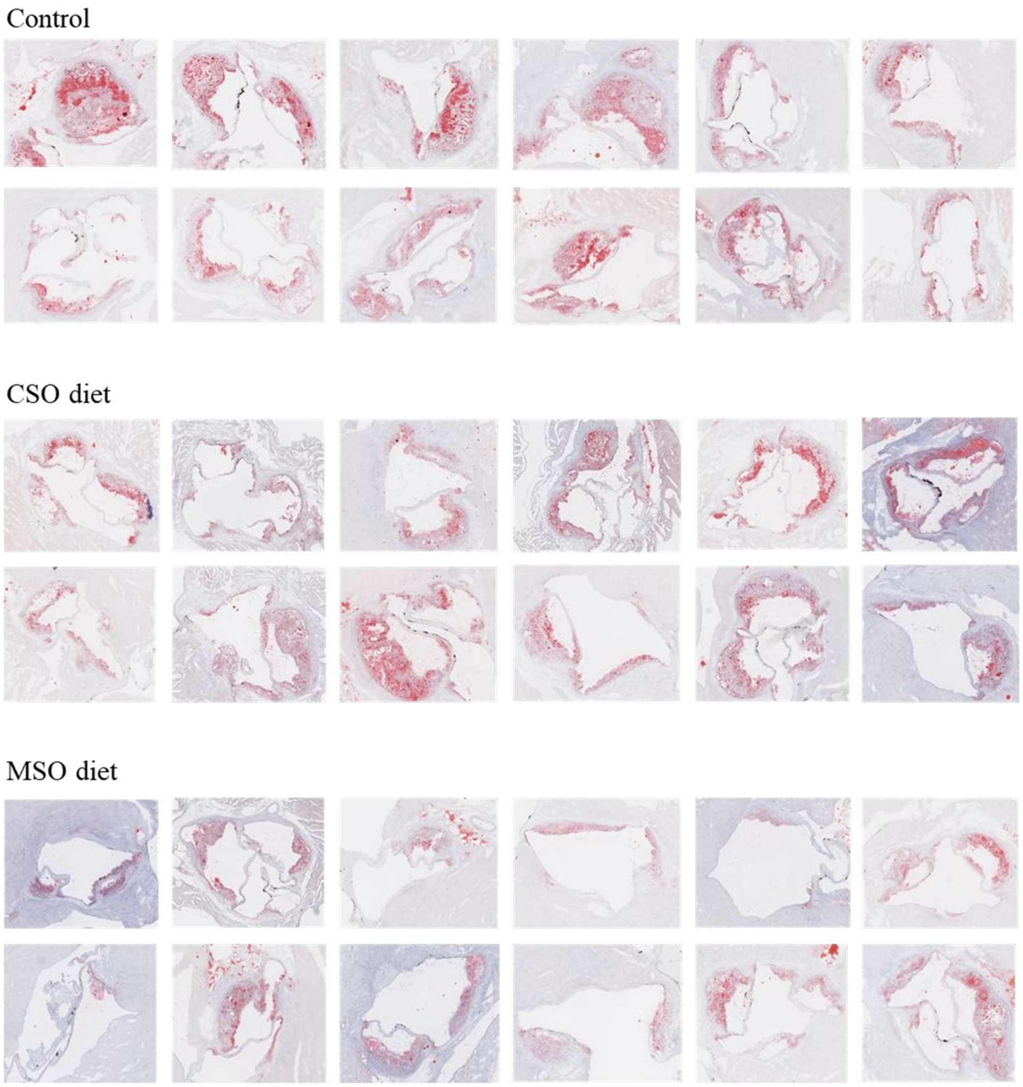

Supplemental Figure S4. Effects of dietary CSO and MSO for 12 months on atherosclerosis and aortic calcification in LDLR-KO mice. Mice (n = 3/group) were fed a Western diet supplemented with 5% (w/w) CSO, MSO, or none (control) for 12 months. (A) Representative *en face* Sudan IV staining of aorta, (B) Quantitative analysis of Sudan IV-positive plaque area of aorta, and (C) Calcium score of aorta. CSO: conventional soybean oil, MSO: modified soybean oil. Values represent the mean ± SEM. * $p < 0.05$ compared with the control Western diet; # $p < 0.05$ compared with the CSO diet.

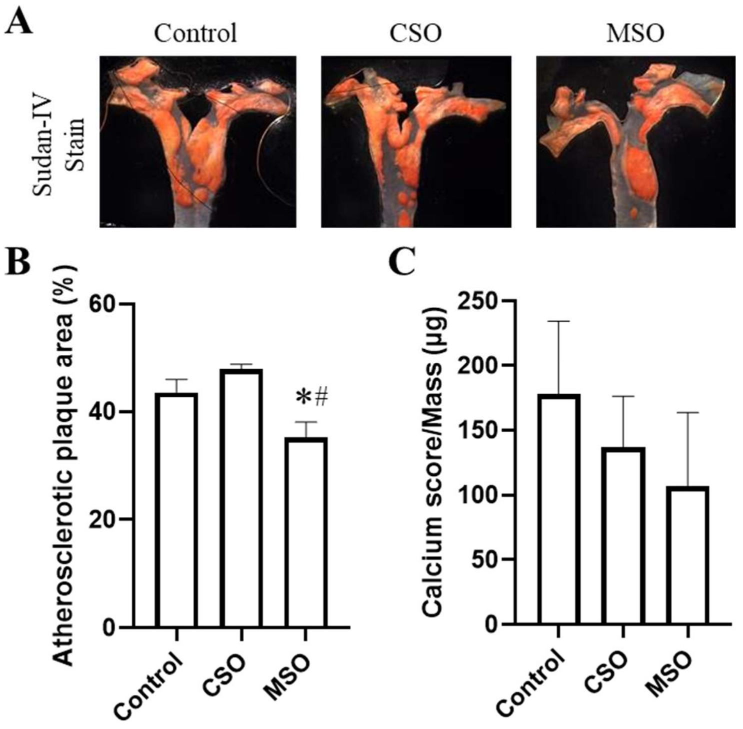

Supplemental Figure S5. Effects of dietary CSO and MSO on plasma cytokine levels in LDLR-KO mice. Mice (n = 3/group) were fed a Western diet supplemented with 5% (w/w) CSO, MSO, or none (control) for 12 weeks. CSO: conventional soybean oil, MSO: modified soybean oil. Values rep-resent the mean ± SD. * $p < 0.05$ compared with the control Western diet; # $p < 0.05$ compared with the CSO diet.

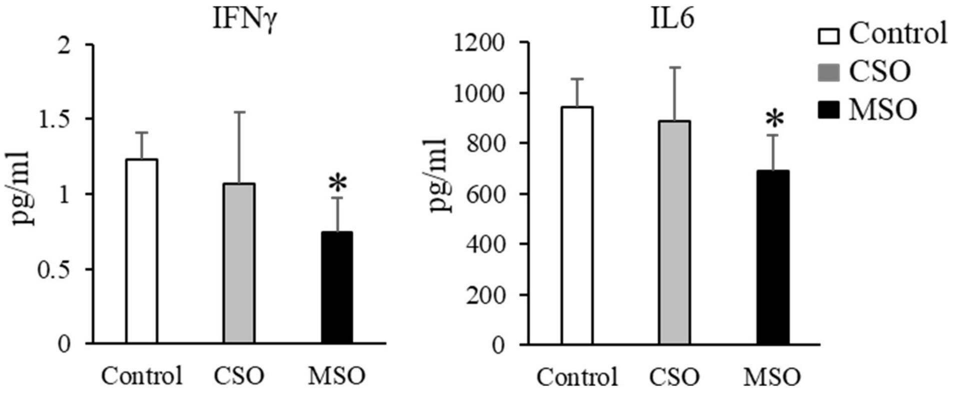

Supplement: Supplementary file 1 [file ijms-23-08385-s001.zip › ijms-1815446-supplementary.pdf]
